# Overweight in Domestic Cats Living in Urban Areas of Italy: Risk Factors for an Emerging Welfare Issue

**DOI:** 10.3390/ani11082246

**Published:** 2021-07-30

**Authors:** Laura Arena, Laura Menchetti, Silvana Diverio, Giovanna Guardini, Angelo Gazzano, Chiara Mariti

**Affiliations:** 1 Laboratory of Ethology and Animal Welfare (LEBA), Department of Veterinary Medicine, Perugia University, 06126 Perugia, Italy; laurarena@hotmail.it (L.A.); laura.menchetti7@gmail.com (L.M.); 2 Department of Veterinary Sciences, University of Pisa, 56124 Pisa, Italy; giovanna.guardini@gmail.com (G.G.); angelo.gazzano@unipi.it (A.G.); chiara.mariti@unipi.it (C.M.)

**Keywords:** domestic cat, obesity, overweight, risk factors, cat management, welfare

## Abstract

**Simple Summary:**

Overweight and obesity are among the most important health problems in domestic cats. These conditions can be related to several diseases in cats and can influence their general welfare. In the present study, 197 cat owners attending veterinary clinics for routine visits completed a questionnaire focused on their cat demographics, management, environment, dietary habits and behavior. On the same occasion, a researcher assessed the body condition of each cat to determine whether they were underweight, normal or overweight. The cat body condition was statistically related to many of the factors explored by the questionnaire. For instance, age higher than 7 years, neutering, being left alone all day and being stressed were positively associated with overweight cats. Conversely, the presence of other animals was negatively associated with overweight. The identification of the risk factors for feline overweight allows veterinary practitioners and behaviorists to give cat owners appropriate advice on cat management and housing conditions. As a consequence, cat overweight-related risks may be reduced and animal welfare improved.

**Abstract:**

Overweight is common in cats and has health and welfare implications. This study aimed to assess potential predictive/protective factors for feline overweight associated with owner management and their relationship with cat behavior and welfare. A questionnaire was administered to 197 owners to collect information about cat demographics, management, environment, dietary habits and behavior. The feline Body Mass Index was recorded for each cat. Univariate logistic regression was used to evaluate the association of parameters with overweight cats. Variables with a *p*-value < 0.10 at univariable analyses were selected for the multivariable model. Most cats were mixed- breed, 1–7 years old and neutered; 51.3% were overweight. Age higher than 7 years, neutering, being alone all day and being stressed were predictive for overweight (*p* < 0.05). Conversely, the presence of other animals had a protective effect (*p* < 0.05). A general disagreement between owners’ perception and clinical evaluation of cats’ body condition was a common and significant risk factor for overweight (OR = 8.532, 95% CI = 4.073–17.875; *p* < 0.001). According to the owners, many veterinarians did not inform them about their cat being overweight nor about the risks (*p* < 0.001). This study provides helpful information on the influence of management and environment on cats’ body condition and its relationship with cat welfare.

## 1. Introduction

Overweight and obesity have been defined as an excess of adipose tissue in an animal’s body [1]. These conditions reflect, respectively, a surplus of 15% and 30% of the ideal body weight [2,3]. Obesity has been reported to be the most common nutritional disorder in companion animals [4,5] and the second health problem after dental diseases in domestic cats [6]. In the literature, the prevalence of overweight or obesity is reported to involve up to 63% of the cat population [7,8,9,10,11]. Among these studies, a wide variation in methodology probably leads to such variety. 

There are numerous techniques available for assessing body condition in cats. These can be classified in: (a) clinical assessment, such as body weight, body condition score (BCS) and morphometric measurements such as the body mass index (BMI); (b) research aimed assessments, such as bioelectrical impedance analysis and dual-energy x-ray densitometry, computed tomography and magnetic resonance imaging [12,13].

Practical clinical assessments are of particular interest because of their high applicability. Body weight is the simplest parameter to measure body condition. However, its main limitation is that it is not able to differentiate the volume body, in other words between a sizeable lean cat and an obese small one. BCS is probably the most used and known technique [2,4,14]; this method estimates the cat body condition by visually ranking it in predefined categories, ranging from “emaciated” to “severely obese”. Alternatively, the morphometric analysis consists of taking body measures that, in cats, correlate well with either lean body mass, such as the length of the head, thorax and limbs [15], or with fat body mass, such as the thorax circumference [16]. The BMI is a validated technique, which can be used in clinical practice to show the owners the body condition of their cats [12,17]. 

Several authors state that the prevalence of feline obesity is increasing in parallel with obesity in humans [4,18,19,20,21]. The trend is worrying because overweight and obesity contribute to the aetiology of many serious illnesses and may jeopardize the overall quality of life in both humans [19,22,23] and animals [24], as well as contributing to reduced life expectancy [4,13,25,26,27]. In cats, obesity is associated with several problems such as reduced insulin sensitivity and diabetes mellitus, hepatic lipidosis, degenerative arthritis and lameness, development of urinary tract diseases and non-allergic skin conditions [4,21,28,29,30,31,32]. 

In order to limit overweight in cats, it is essential to identify more precisely those contributing environmental factors that can be altered and/or controlled. In humans, the increasing prevalence of overweight and obesity is due to environmental factors and habits. [33,34,35]. In cats, we assume that some factors, such as individual attitudes and behavior, food intake and physical and play activities, may have a crucial role. In addition, since pets are primarily dependent on their owners’ attitudes, believes and lifestyle, we can suppose that owners’ routine management can influence the body condition of their cats. Previous studies identified specific factors contributing to the occurrence of obesity and overweight in cats [6,28]. Intrinsic and unmodifiable factors, such as gender, age and neutered status, as well as reversible management and environmental factors, such as the lack of activity and indoor housing [9,36,37], overfeeding [38] and dietary supplementation [39], have been consistently described in previous studies. On the contrary, conflicting findings have been reported for ad libitum feeding [4,39,40,41].

It is essential to gain further knowledge of the predisposing factors to identify cats at risk of overweight and obesity at an earlier stage, enabling preventive measures and hindering the development of obesity-related diseases. Furthermore, differences and similarities in obesity-related comorbidities between people and cats provide opportunities for interdisciplinary research.

This study aimed to assess potential predictive/protective factors of overweight in cats associated with owner management and care. Furthermore, we assessed the relationship between overweight and cat specific behaviors and investigated potential welfare implications. 

## 2. Materials and Methods

### 2.1. Subjects

For the present study, both cats and cat owners were involved. Healthy cats over one year of age and their respective owners were recruited from eight veterinary clinics at the moment of the generic/vaccination visit. The clinics were distributed across the Italian territory (four in the North, three in the Centre and one in the South). 

For each cat, a researcher collected the FBMI (Feline Body Mass Index) measurements and some anamnestic and management data by administering a questionnaire to the owners. The person accompanying the pet to the clinic (hereafter the owner) was interviewed after having checked that that person was familiar with the cat and well informed about the cat management and behavior.

The study was conducted in accordance with the Declaration of Helsinki. All cat owners were debriefed about the aim of the study, and they had to provide their informed consent for inclusion in the study. The research was conducted without subjecting the animal to any kind of stress or suffering, during a veterinary visit, and therefore did not require a specific approval from the ethics committee (as provided by Italian Legislative Decree 26/2014, implementing EC directive 63/2010).

### 2.2. Clinical Assessment

For each cat, the researcher used a tape to measure the thoracic circumference at the level of the ninth rib and the distance from the patella to the calcaneal tuber, known as the leg index measurement (LIM). Those measures allowed to calculate the FBMI that is necessary to assess the actual body condition of the animal (underweight, normal or overweight) [12,15]. Details on the method for calculating the FBMI are reported in Figure S1. 

### 2.3. Questionnaire 

The questionnaire was developed in seven sections.

The first section included the FBMI information collected by the researcher; this was the only section filled in by the researcher. 

The second section focused on cat demographic characteristics and was composed of eight closed-ended questions (age, breed, sex, weight, neuter status and possible behavioral changes related to neutering, age at acquisition, origin of the cat, e.g., stray, adopted, born at home etc.).

The third section collected information on the cat living environment and the presence of environmental enrichment through 17 close-ended questions (e.g., family composition, presence of children and other pets, presence of indoor/outdoor areas, health status and medical history, daily habits, elimination and scratching areas, number of hours per day the cat is usually left alone).

The fourth section included 17 close-ended questions about dietary habits and behavior in relation to feeding: type, quantity and quality of food, feeding regime, if the cat asks for food, location of food bowls, possible changes in feeding regime, availability of grass, etc. 

The fifth section comprised questions about play behavior: five closed-ended questions on whether the cat plays in the first place, play areas, time spent playing with people and other animals, number and type of objects used as toys. 

The sixth section contained four closed-ended questions about the presence of aggressive behavior in the cat and, if present, in which situation and towards whom it was exhibited. Owners were also asked to report if they had the impression that the cat was nervous or stressed. 

The seventh section included five closed-ended questions related to the cat body condition, e.g., the owners’ personal opinion about the body condition of their cat, scored by using the 5 point-scale BCS chart included in the questionnaire, which includes a picture and a visual and palpable description for each score (for details see Figure S2) [2]; if the owner monitored the weight of the cat; if the vet ever noticed the possible overweight/obese status of the cat; if the owner had ever taken measures; and if the animal displayed changes in the behavior after having gained weight. 

Providing an answer to all questions was not compulsory for the participants.

### 2.4. Statistical Analysis

The data obtained from the questionnaires were first entered into an Excel spreadsheet and then transferred into the statistical program SPSS Statistics version 25 (IBM, SPSS Inc., Chicago, IL, USA) for analysis. The level of statistical significance was set at <0.05.

Distributions within categorical variables were analyzed using Chi-square goodness of fit tests. The agreement between owners’ personal opinion about the body condition of their cat and the classification of the expert (overweight or not-overweight) was evaluated through McNemar’s test [42].

First, univariate logistic regressions were used to evaluate the association of each analyzed parameter with overweight [43]. The dependent variable was “Overweight”, where “Not overweight” was the reference category. Then, a multivariable logistic regression using forced enter methods were built to identify the factors independently related to overweight, adjusting for the other variables. For this model, the variables were selected in accordance with their significance to the univariate analysis (*p*-value < 0.10) and their reciprocal associations in order to avoid multicollinearity problems and in accordance with the principle of parsimony (i.e., the variable “*Where the cat sleeps*” was excluded because associated with “*Where the cat lives*”; the variable “*How much the cat plays with other animals*” was chosen because it had the highest odds ratios (OR) among the questions related to the cat play behavior). Multicollinearity was checked by using a tolerance and Variance Inflation Factor [44]. The OR with 95% confidence intervals (CI) and *p* values from Wald statistics were reported to indicate the strength of the associations.

## 3. Results

### 3.1. Cat Population

The total number of cats assessed and owners interviewed was 197. 

Demographic characteristics and main management aspects of the cats are detailed in Table S1. Most cats were mixed breed (78.7%; *p* < 0.001) and between 1 and 7 years old (56.3%; *p* < 0.001). There was no substantial difference between males (45.5%) and females (55.5%), but most were neutered (91.4%; *p* < 0.001). Most of the cats had been adopted from the street (43.1%) or came from another house (24.4%; *p* < 0.001), lived at home with other animals (79.2%; *p* < 0.001)*,* and were free to roam inside and outside the house (62.8%; *p* < 0.001). On the basis of the FBMI measurements, cats were 48.7% (*n* = 96) normal and 51.3% (*n* = 101) overweight. No cats were found to be underweight.

### 3.2. Association between Overweight and Factors Related to Demographic Characteristics, Management and Behaviour of Cats: Results from Univariate Analysis

**Figure 1:**
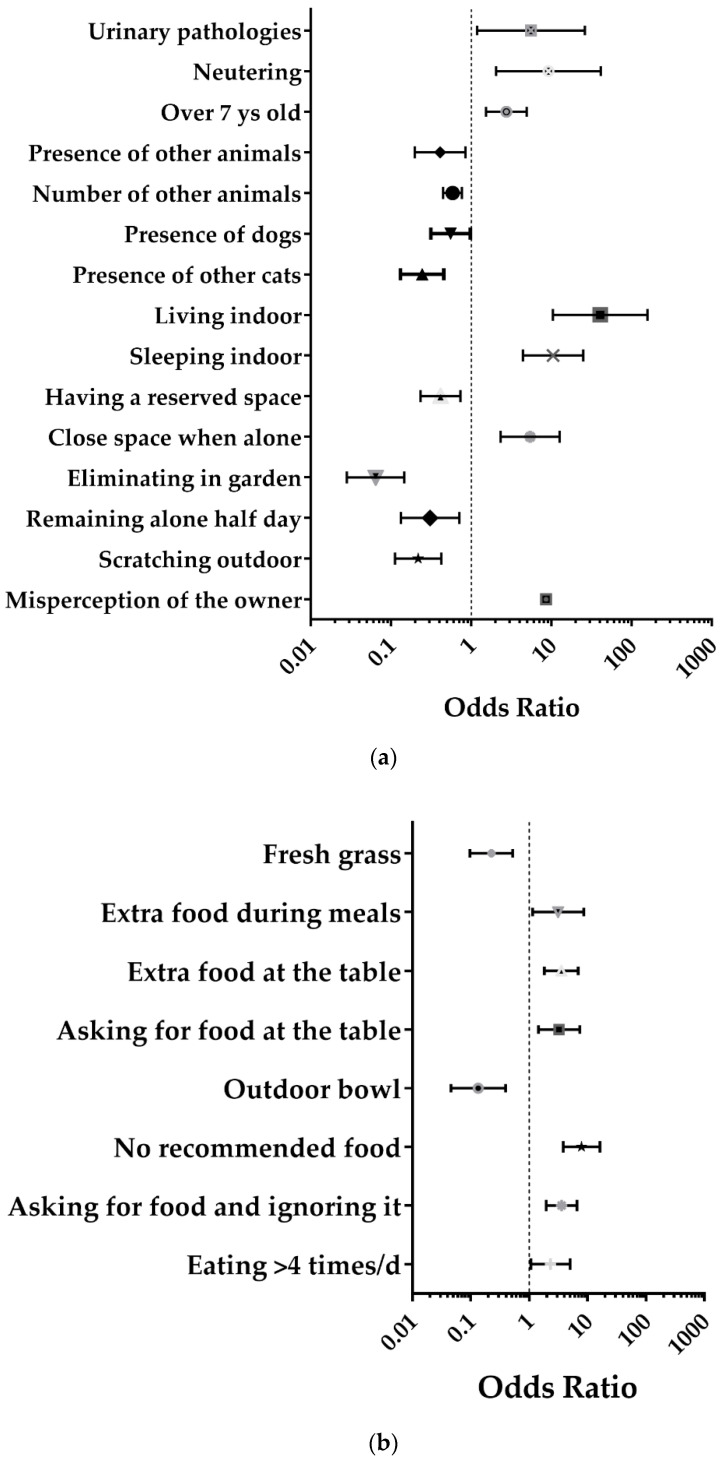

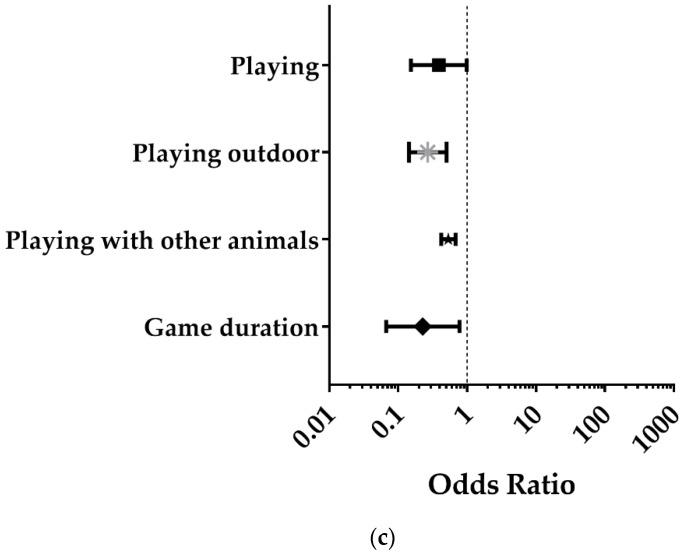
Forest plots of the parameters significantly associated with overweight: results of the univariate analysis (unadjusted OR and 95% CI) for factors related to demographic characteristics and general management of cat (**a**), food management (**b**) and cat behavior (**c**). The detailed description of the variables and the reference categories are shown in Tables S2–S4.

Factors significantly associated with overweight from the univariate analysis are shown in Figure 1, while detailed results, including not significant values, are shown in Tables S2–S4. 

#### 3.2.1. Factors Related to Demographic Characteristics and General Cat Management 

The following parameters were positively associated with the risk of overweight: age (*p* < 0.01), neutering status (*p* = 0.004), urinary disorders (*p* < 0.05), living (*p* < 0.001) and sleeping indoors (*p* < 0.001) and being enclosed in a room at home when alone (*p* < 0.001). Furthermore, the owner’s misperception of the cat’s body condition was positively associated with the occurrence of overweight in cats (<0.001). 

Conversely, the following factors were found to be protective against overweight: the presence of other cats (*p* < 0.001) and dogs (*p* < 0.05), with the risk of overweight decreasing as the number of animals in the household increases (*p* < 0.001), availability of a secluded space that the cat uses as a refuge (*p* < 0.01), eliminating in the garden compared to the litter box (*p* < 0.001), scratching objects outdoors (*p* < 0.001), staying alone at home half-day compared to the whole day (*p* < 0.01).

#### 3.2.2. Factors Related to Food Management

The risk for overweight was higher in cats that asked for food and then ignored it (*p* < 0.001), asked for food while the owner was eating (*p* < 0.001), if the owner provided extra food during his/her meals to the cat (*p* < 0.05) if the owner provided amounts of food more than recommended (*p* < 0.01) or if the owner did not know what the proper amount was (*p* < 0.001) and if cats eat more than 4 times a day (*p* < 0.05). 

Protective factors related to food management were: placing the bowl outside the home (*p* < 0.001) and availability of fresh grass (*p* < 0.01).

#### 3.2.3. Factors Related to Cat Behavior

The risk of overweight decreased if the cat played (*p* < 0.05), played outside (*p* < 0.001), played with other animals (*p* < 0.001) and if playing sessions lasted several minutes instead of a few seconds (*p* < 0.05).

### 3.3. Association between Overweight and Factors Related to Cat Management and Behaviour: Results from Multivariate Analysis

**Figure 2:**
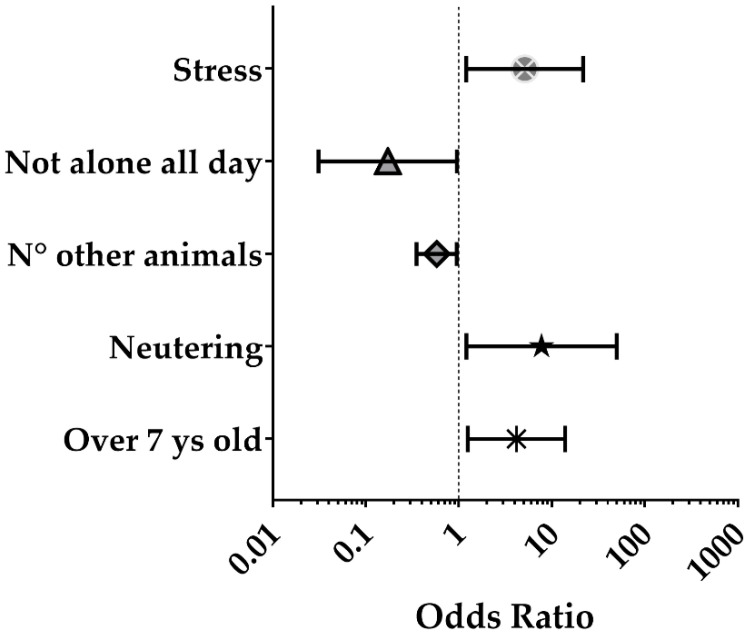
Forest plot of the parameters significantly associated with overweight: results of the multivariable analysis (adjusted OR and 95% CI). The detailed description of the variables and the reference categories are shown in Table S5.

The independent factors predicting overweight, as a result of multivariable analysis (Figure 2 and Table S5), were an age higher than 7 years (*p* < 0.05) and neutering (*p* < 0.05); the risk for overweight also increased if the cat remained alone in the house all day (*p* < 0.05) and if, according to the owner, the cat seemed stressed (*p* < 0.05). Conversely, the presence of other animals was a protective factor, with a greater effect as the number of animals (both dogs and cats) in the household increased (*p* < 0.05).

### 3.4. Owners Perception of Overweight Cats 

Most owners did not monitor the weight of their cat (*n* = 119, 60.4%; χ^2^= 8.5; *p* < 0.01) even though half of them (*n* = 60, 50.4%) had an overweight cat. Bodyweight was monitored in 41/101 (40.6%) overweight cats.

The majority of cat owners (*n* = 152, 77.6%) declared that the veterinarian did not tell them that their cat was overweight (χ^2^= 59.5; *p* < 0.001; Table 1). However, over 40% of them (*n* = 65) had an overweight cat. Furthermore, there was no agreement between the response of overweight cat owners to the item “Is in your opinion your cat obese or overweight?” and the classification by FBMI scoring (McNemar’s test: *p* < 0.001). Less than half of the overweight cat owners were aware of their cat’s overweight (48/101, 47.5%).

The lack of owner’s awareness of the actual status of their cat was a significant risk factor for overweight (OR = 8.532, 95% CI = 4.073–17.875; *p* < 0.001; Figure 1a).

Based on the scores assigned by the owner to BCS, 60/197 cats were overweight, but nine of them were erroneously classified, being of average weight. Conversely, 50/101 owners of overweight cats assigned to their cats lower BCS scores than the cat’s actual physical condition assessed by the FBMI. There was no agreement between the classification made using FBMI and the score given by the owner to the BCS of his cat (*p* < 0.001).

## 4. Discussion

The study aimed to assess the relationship between owners’ daily cat management and care and cat body condition. It also aimed to investigate the relationship between feline overweight, specific cat behaviors and cat welfare. The study was carried out through questionnaires completed by owners of 197 adult cats visiting eight Italian veterinary clinics for routine clinical examinations. Among the cat individual characteristics, middle age and neutered status were confirmed as major risk factors for feline overweight. Both these factors are independent predictors, as demonstrated by the multivariate analysis. 

Middle/adult age (over 7 years old) appears to be related to overweight. A peak in risk in this age range was also found by other authors [9,11,36,39,45,46,47,48,49,50,51,52]. The reduction in risk seen in geriatric cats [9,11,39,47,51,53] is probably due to concurrent geriatric diseases, such as feline hyperthyroidism [53] and chronic kidney disease [54]. It is also possible that owners increase their attention to the cat’s health as the latter grows older. On the contrary, early age may positively influence other factors such as activity level, playing behavior and emotional state.

Conversely to other studies, where male cats were more at risk of being overweight, we did not find a difference based on the cats’ sex. Neutering, instead, was found to be a strong predictor. Although overweight can occur in both neutered and intact animals and is influenced by a number of factors, such as feeding management and cats’ activity level, previous literature suggests that significantly more neutered animals are overweight compared to intact animals [49,55,56]. Soon after spaying, most cats show a significant increase in daily food intake, significantly lower energy requirements for maintenance and a decrease in physical activity [57], leading to body weight gain in the form of fat mass. After gonadectomy, total fat mass increases by approximately 40%–120%, compared with about 10%–13% of lean mass [41,57]. In the current study, we investigated whether neutering was linked to changes in the cat behavior; although data are not reported here, owners declared that their cats showed a decrease in physical activity after gonadectomy, which is likely to be responsible for weight gain, as suggested by Belsito et al. [57].

Good management of cat weigh is important because of its association with an increased risk of other medical problems [58]. In the current study, the correlation between overweight and the occurrence of urinary tract diseases resulted from the univariable analysis, not confirmed by the multivariable analysis, as proof of the potential multifactorial aetiology of this health problem. Still, most of the previous studies on this topic have shown a positive correlation between these two conditions [5,9,11,29,48,58], with lower urinary tract diseases such as urethral obstruction commonly seen in overweight, neutered, middle-aged male cats [59]. Although we did not detect a wide range of health problems other than urinary tract diseases, in previous literature, overweight in cats is related to several other pathological conditions, such as arthritis, dermatopathy, diabetes mellitus, hepatic lipidosis, neoplasia, gastrointestinal, cardiac, musculoskeletal and oral diseases [9]. All these associations clearly show the link between overweight and the welfare of cats, especially in relation to physical health.

The environment in which cats live might also influence their body condition. In our study, the presence of other animals, both dogs and cats, was protective for the occurrence of overweight, confirming previous literature [7,8,39,47], with a minor risk when the number of animals increases. The multivariable statistical model reveals that this is also an independent factor. However, other researchers did not find that living without other animals was a significant risk factor [5,6]. The presence of other animals is probably responsible for differences in food management. We can suppose that people who own both cats and dogs may be less inclined to provide premium varieties of cat food, especially if there is a scavenging dog in the household. Allen and colleagues [8] suggested that in multispecies households, dogs may intimidate cats while they are eating and drive them away from their food, thereby limiting cats’ calorie intake. An additional possible explanation is that cats and dogs may be playmates, thereby increasing the level of physical activity. In support of this, our results show that playing with other pets exerts a protective effect against overweight. For the same reason, playing, in general, seems to be protective factor. 

Previous studies have identified inactivity as a risk factor for feline overweight and obesity [8,48], and increasing physical activity through toys and playing sessions is a useful adjunct to dietary therapy [4]. However, cats are generally regarded as sedentary animals, with occasional moments of running and playing. Thus, a minority of cat owners play with their cats, and playing occurs mainly for owner enjoyment and to facilitate social interaction with the cat rather than as a means to foster exercise [60]. Exercise is a key factor in health for several reasons: it increases the metabolic rate, energy expenditure, fat oxidation and fat loss may assist in lean tissue preservation and strengthen muscle tissues may help prevent the rapid regain in weight that can occur after successful weight loss promotes cardiovascular health provides mental stimulation and improves the overall quality of life [61,62]. Playing also allows cats to exhibit their natural hunting behavior, which is thought to release endorphins, just as they are released in humans during exercise [63], consequently improving welfare of cats.

In accordance with our results, Kienzle et al. [50] showed that owners of overweight cats played less and more often used extra food with their pets, whilst owners of normal cats more often used extra playtime as a treat. This suggests that cat-owner interactions should be addressed by veterinarians as part of a weight management program by explaining to the owner the benefits of playing with the cat, and educating them on play as a better form of reward. This advice needs to be tailored to the individual cat and the situation: toys may work well for some cats, cat trees or play stations are excellent for others, and not every cat responds well to interactive toys that reward him/her with food. 

From our results, among environmental factors, living and sleeping exclusively indoors was found to be a predictor for overweight. Conversely, living both indoors and outdoors and spending time outdoors performing various activities, such as eliminating, eating, hunting, scratching and playing, appear to be protective factors against overweight. Some studies have already reported indoor confinement as a risk factor for overweight [7,8,39,45,47], whereas others have failed to find such association [5,64]. If feasible, protected outdoor activity should be encouraged, as it is an excellent stimulus for play and exercise in cats. In fact, indoor cats tend to be more sedentary, which has detrimental effects on their physical and psychological health and well-being [13].

Lifestyle modification and environmental enrichment are the most important additions to any program for overweight and obesity prevention or management [65]. Our findings suggest that indoor cats that are prevented from entering and exploring certain rooms have a higher probability of being overweight compared to cats that are let free to roam and have outdoor access. Ensuring that cats have an adequate space is important for the prevention of overweight and for their overall welfare. Moreover, from our results, cats that are provided with a secluded area that they can use as a refuge are less prone to become overweight. Having a hiding place is vital part of an environmental enrichment program for cats [66], in addition to multiple and separated key environmental resources such as food, water, toileting areas, scratching and resting areas, opportunity for play and predatory behavior, positive, consistent and predictable human–cat social interactions [67].

Nutritional enrichment is also part of environmental enrichment. For instance, for cats, eating fresh grass is an important species-specific behavior. From our results, allowing the cat to have fresh grass available for consumption is a protective factor for overweight. On the one hand, we can assume that having fresh grass is important in itself, and attentive owners can provide it as environmental enrichment. On the other hand, this data could be influenced by the possibility for cats to have access to an outdoor space. Therefore, presumably for this reason, this variable was not significant for the multivariate analysis. 

From our results, staying alone at home few hours (up to half-day) compared to the entire day is protective against the occurrence of overweight in cats. From the multivariate model, it was found as an independent factor. As mentioned above for the companionship of other pets, social interactions and playing activities seem particularly important (and possibly underestimated) in the prevention of cat overweight. 

Possible emotional and behavioral factors may be involved in the development of overweight such as anxiety, depression, failure to establish a normal feeding behavior and to develop control of satiety [4,68]. From our results, owners of overweight cats reported their cats to be more stressed compared to normal cats. This result appears to be an independent predictor. While stress is an accepted cause of overeating and subsequent weight gain in people and laboratory animals, the impact of stress on cats has not been well studied [65]. In literature, it is also described that cats, after a weight loss treatment based on caloric restriction and feeding regimen changes, cats are significantly more likely to have increased affiliative and affective behavior towards their owners, such as purring, sitting and resting on the lap. This may explain why we found an association between being stressed and being overweight in cats. In fact, the perception of stress in cat owners can vary according to their knowledge about feline ethology, with social avoidance being a relevant factor for some of them [69]. Taken together, our results confirm that overweight is related to both physical and psychological aspects of cat welfare. 

It is proved that feeding management has a crucial role in the occurrence of feline overweight. Downes et al. [60] suggest that offering human food to cats during mealtimes or while food is being prepared is a common habit, and it is considered as being part of the owner-pet relationship. However, in accordance with other studies [4,60], our findings highlight that giving cats extra food is a risk factor for overweight. Similarly, food-begging behavior is related to overweight in cats [70]. Furthermore, from our results, most of the cats that beg for food, once it is provided by the owner, may ignore it. This factor is positively related to the occurrence of overweight. We suggest that cat behavior could be misconceived from the owners’ perspective [43]. We can hypothesize that most of the times, the cat vocalizations and/or rubbing behavior may reflect a motivation for social interaction rather than food. In fact, Kienzle and Bergler [50] reported that owners who respond to such behaviors with play rather than food treats are more likely to have normal weight pets. When setting up weight management protocols, veterinarians should include information on cat social motivation and behavior, as well as on possible owner’s responses that do not involve food. 

In this study, we found an association between feeding more than four meals a day and being overweight. However, some previous studies showed conflicting results, with both ad libitum feeding and being fed twice daily as risk factors [47,64]. In our study, the free choice basis as food allowance did not appear as a risk factor, in accordance with some previous studies [5,47], but in contrast with others [41,47,71]. Our results suggest that the total amount of food, more than the number of meals may be important in weight control. In fact, we found that owners who administered an amount of food different from that suggested by the label had cats at higher risk of developing overweight. Colliard and colleagues [5] found that, even if the quantity of food administered to the cat is prescribed by a veterinarian or read on the food label, owners mainly do not weigh it. Veterinarians should spend more time educating owners on the possible risks for not following official nutritional advice. As described above, giving the cat the possibility to consume the meal outdoors and locating the food bowl outside are protective against the occurrence of overweight. This factor may be generally associated with the possibility for cats to increase their overall physical activity. German [4] suggests that cats can also be encouraged to work for their food by using feeding toys.

In the current study, most owners were not able to discriminate the actual body condition of the cat. In fact, the number of cats reported to be overweight by their owners using the BCS was lower compared to the number of overweight cats detected by the researcher using the FBMI. It is described in the literature that there is a general mismatch between professional-recorded and owner-recorded cats’ body condition [5,46]. The tendency of owners to underestimate the BCS of their pet [72,73] probably leads to the lower prevalence seen in studies based on owners’ perception and assessment of the pet body condition [5,6,8,46,64]. In the current study, as previously described, it has been shown that owner underestimation of the cat’s BCS is in itself a risk factor for overweight [5,6,8,74,75]. Effective communication and treatment strategies should be designed to engage clients to successfully combat this widespread problem [73]. Instead, the vast majority of owners of overweight cats declared to have not been advised by the veterinarian about the body condition of their pets. This is quite unexpected, because overweight could predispose cats to health problems and veterinarians should act to prevent them. It is possible that veterinarians have different attitudes towards this problem or even that owners tend not to pay enough attention to their recommendations because they do not like to be told their cat is overweight. These findings remark the importance of the role of veterinarians in advising owners on the physical conditions of their pets, stressing the importance of being aware not only about the body condition but also of the effect that overweight may have on the health, welfare and lifespan of their cats.

### Limitations of This Study

Although the number of respondents is relatively high, the sample size of this study cannot be regarded as representative of the Italian feline population. In fact, we only considered a healthy population of cats and all the clinics were located in urban areas. Furthermore, as our research focused on overweight cats with voluntary participation, we cannot exclude that the participation in the study by owners was facilitated in those who had an overweight cat. Maybe due to these characteristics of the methodology, the high prevalence (51.3%) of overweight found in this study is one of the highest reported in the literature. Depending on the study, the prevalence of obese or overweight cats varies within a wide range (6–52%) [5,6,8,9,10], with practitioners estimating even higher numbers compared with the self-reported estimation of the body condition of cats by owners 5,46]. Furthermore, in our study owners reported lower number of overweight cats compared to researchers; however, it must be highlighted that they used different tools, respectively BCS and FBMI, thus a direct comparison was not possible.

## 5. Conclusions

The present study allowed us to identify several risk factors that can be potentially manipulated to avoid the risk of feline overweight. These include supplying extra food to cats without considering the total calorie intake and providing them with an amount of food different from what suggested in the label. Other risk factors identified by this study are less amenable to change, such as age, neutering status and the availability of outdoor space. On the contrary, playing activity, the possibility to explore the whole house and less time spent alone at home were identified as protective factors. 

These results can be used to suggest evidence-based preventive strategies for feline overweight and obesity, and so they can potentially help reduce the prevalence of this widespread problem. Additionally, understanding owner behavior allows for a more targeted approach to prevent and treat cat overweight.

According to the owners’ report, veterinarians did not tend to discuss cats’ body conditions with them, even if the cat was overweight. Furthermore, there was a low agreement between the owner-reported body condition and the assessment performed by the researcher, with owners tending to underestimate their cats’ overweight. Veterinarians should dedicate part of the routine visits to talk about the cats’ body condition with owners, with particular attention to that category of animals at higher risk, such as adult, neutered and indoor cats. Owner education and awareness must be encouraged, and veterinarians play a crucial role in this. Veterinarians should suggest to owners to use forms of reward other than feeding treats, such as play, and to increase the environmental enrichment for cats. Surveillance and monitoring of the health of cats are especially relevant in light of the current epidemic of human obesity and in light of the altered human perception of what is actually a normal body condition for cats.

## Figures and Tables

**Table 1:** Opinion of the owner about the weight of their cat.

Question		Number	Percentage	χ^2 #^	*p*-Value ^#^
The vet said that your cat is overweight or obese	No	152 *	77.6%	59.5	<0.001
	Yes	44	22.4%		
You have taken action	No	23 *	51.1%	19.4	<0.001
	Hypocaloric diet	12 *	26.7%		
	Reduction of food quantity	6	13.3%		
	No moist/homemade diet	4	8.9%		
You monitor your cat’s weight	No	119 *	60.4%	8.5	0.003
	Yes	78	39.6%		
Where you monitor the weight of your cat	Vet	44 *	56.4%	24.9	<0.001
	Home	26	33.3%		
	Both	8	10.3%		
In your opinion, your cat is overweight or obese	No	138 *	70.1%	31.7	<0.001
	Yes	59	29.9%		
According to the BCS assigned by the owner the cat is overweight	No	137 *	69.5%	30.1	<0.001
	Yes	60	30.5%		
In your opinion, which is the BCS of your cat	1	2	1.0%		
	2	36	18.3%		
	3	98 *	49.7%		
	4	52 *	26.4%		
	5	9	4.6%		
In your opinion, overweight and obesity can be a health problem for your cat	No	21	10.7%	157.2	<0.001
	Yes	148 *	75.5%		
	I do not know	27	13.8%		
If put on weight, your cat does the things it did before	No	42 *	43.8%	4.8	0.093
	Yes	28	29.2%		
	I do not know	26	27.1%		

**^#^** results of Chi-square goodness of fit test, * higher observed number with respect to expected (all categories equal).

## Data Availability

Data are available on request.

## Supplementary Materials

The following are available online at https://www.mdpi.com/article/10.3390/ani11082246/s1, Figure S1: Feline Body Mass Index (FBMI) chart used by the researcher, Figure S2: 5 point-scale BCS chart included in the questionnaire for cat owners’ self-assessment on the body condition of their cats, Table S1: Demographic characteristics and general management of the cat, Table S2: Association between overweight and factors related to demographic characteristics and general management of cat. Results of univariate logistic regression analysis, Table S3: Results of bi-variable logistic regression analysis. Association between overweight and factors related to food management, Table S4: Results of bi-variable logistic regression analysis. Association between overweight and factor related to playful and aggressive behaviors, Table S5: Independent predictors of overweight. Results of multivariable model.
