# Supplementary material for: Overweight in Domestic Cats Living in Urban Areas of Italy: Risk Factors for an Emerging Welfare Issue
Source: Animals (Basel). 2021 Jul 30;11(8):2246. doi: 10.3390/ani11082246 (PMC8388420; doi:10.3390/ani11082246)

Figure S2: 5 point-scale BCS chart included in the questionnaire for cat owners' self-assessment on the body condition of their cats.

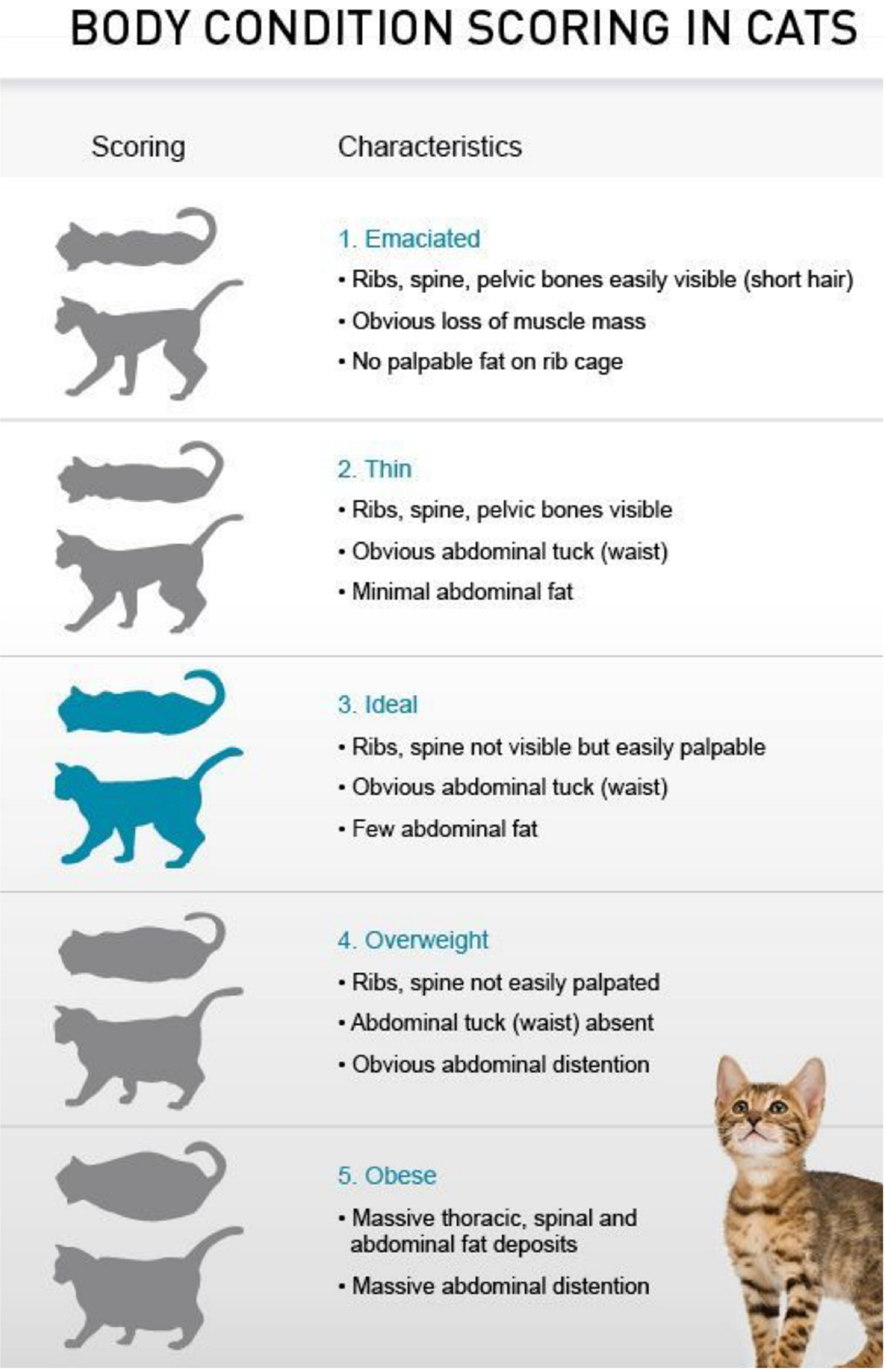

Supplement: Supplementary file 1 [file animals-11-02246-s001.zip › animals-1281905-supplementary. V2/Figure S2.pdf]
