# Supplementary material for: Overweight in Domestic Cats Living in Urban Areas of Italy: Risk Factors for an Emerging Welfare Issue
Source: Animals (Basel). 2021 Jul 30;11(8):2246. doi: 10.3390/ani11082246 (PMC8388420; doi:10.3390/ani11082246)
Supplement: Supplementary file 1 [file animals-11-02246-s001.zip › animals-1281905-supplementary. V2/Supplementary Material.pdf]

Table S1. Demographic characteristics and general management of the cat

DEMOGRAPHIC CHARACTERISTIC		NUMBER	PERCENTAGE	$\chi^{2\#}$	P value[#]
**Age**	**1-7 years**	111*	56.3%	48.0	<0.001
	**>7-10 years**	49	24.9%		
	**>10 years**	37	18.8%		
**Sex**	**Male**	91	46.2%	1.1	0.285
	**Female**	106	53.8%		
**Neutering**	**No**	17	8.6%	134.9	<0.001
	**Yes**	180*	91.4%		
**Breed**	**Mixed**	155*	78.7%	64.8	<0.001
	**Purebred**	42	21.3%		
**Source**	**Shop**	12	6.1%	138.8	<0.001
	**Breeding**	3	1.5%		
	**Home**	48*	24.4%		
	**Street**	85*	43.1%		
	**Cattery**	16	8.1%		
	**Born in home**	33	16.8%		
**Presence of other animals at home**	**No**	41	20.8%	67.1	<0.001
	**Yes**	156*	79.2%		
**Where the cat lives**	**Outdoors**	31	15.8%	77.3	<0.001
	**Indoors**	42	21.3%		
	**Outdoors and indoors**	124*	62.9%		
**Where the cat sleeps**	**Indoors**	148*	75.1%	49.8	<0.001
	**Outdoors**	49	24.9%		

[#] results of Chi-square goodness of fit test
* higher observed number compared with expected (all categories equal)

Table S2. Association between overweight and factors related to demographic characteristics and general management of the cat. Results of the univariate logistic regression analysis.

PARAMETER	Unadjusted OR	95% CI LOWER	95% CI UPPER	P value
**Age**				
**>7 years vs 1-7 years**	2.738	1.528	4.906	**0.001**
**Sex**				
**Female vs Male**	0.760	0.434	1.334	0.339
**Neutering**				
**Yes vs No**	9.167	2.036	41.261	**0.004**
**Months at neutering**	0.999	0.975	1.024	0.966
**Change of behaviour after neutering**				0.140*
**Yes vs No**	1.867	0.949	3.672	0.070
**I do not know vs No**	0.884	0.365	2.141	0.784
**Purebred**				
**Yes vs No**	1.529	0.765	3.054	0.229
**Source**				0.088*
**Breeding vs Shop**	-[1]	-[1]	-[1]	-[1]
**Home vs Shop**	3.333	0.877	12.666	0.077
**Street vs Shop**	2.722	0.761	9.742	0.124
**Cattery vs Shop**	1.556	0.329	7.361	0.577
**Born at home vs Shop**	1.000	0.246	4.062	1.000
**Age at acquisition**				0.343*
**3 months-1 year vs < 3 months**	1.368	0.688	2.720	0.371
**>1 year vs < 3 months**	0.556	0.176	1.750	0.315
**Number of family members**	0.820	0.664	1.012	0.065
**Presence of children**				
**Yes vs No**	0.671	0.348	1.291	0.232
**Presence of other animals**				
**Yes vs No**	0.408	0.197	0.847	**0.016**
**Presence of other cats**				
**Yes vs No**	0.244	0.131	0.454	**<0.001**
**Presence of dogs**				
**Yes vs No**	0.551	0.313	0.970	**0.039**
**Number of other animals**	0.583	0.445	0.764	**<0.001**
**Relationship with cohabiting cats**				0.259*
**Fear vs main friendly**	-[1]	-[1]	-[1]	-[1]
**Indifference vs mainly friendly**	0.172	0.021	1.430	0.103
**Mainly hostile vs mainly friendly**	0.458	0.138	1.526	0.204
**Relationship with cohabiting dogs**				0.407*
**Fear vs mainly friendly**	0.927	0.295	2.915	0.897
**Indifference vs mainly friendly**	1.405	0.511	3.859	0.510
**Mainly hostile vs mainly friendly**	6.182	0.648	59.003	0.114
**Diseases**				**0.060**
**Urinary vs one**	5.556	1.182	26.111	**0.030**
**Respiratory vs none**	1.667	0.272	10.227	0.581

**Others (gastrointestinal, FIV[2], neoplasia, allergy, otitis) vs none**	3.889	0.785	19.259	0.096
**Surgery**				
**Yes vs No**	2.000	0.658	6.082	0.222
**Where the cat lives**				**<0.001***
**Indoors vs Outdoors**	40.500	10.395	157.795	**<0.001**
**Outdoors and indoors vs Outdoors**	6.641	2.193	20.110	**0.001**
**Where the cat sleeps**				
**Indoors vs Outdoors**	10.444	4.387	24.863	**<0.001**
**Forbidden spaces**				
**Yes vs No**	1.218	0.673	2.205	0.514
**Reserved space**				
**Yes vs No**	0.413	0.233	0.732	**0.002**
**Reserved space and use**				**0.022**
**The cat has a reserved space, but he does not use it vs the cat does not have a reserved space**	0.440	0.185	1.045	0.063
**The cat has a reserved space and uses it as a refuge vs he does not have a reserved space**	0.452	0.243	0.843	**0.012**
**Where the cat eliminates**				**<0.001***
**Garden vs litter**	0.064	0.028	0.146	**<0.001**
**Garden and litter vs litter**	0.222	0.098	0.503	**<0.001**
**Inappropriate elimination**				
**Yes vs No**	0.833	0.426	1.630	0.594
**The cat licks insistently**				0.128*
**Yes vs No**	2.630	0.899	7.691	0.077
**Sometimes vs No**	0.337	.034	3.305	0.351
**Where the cat gets his nails**				**<0.001***
**Outdoors vs only indoors**	0.217	0.112	0.422	**<0.001**
**Outdoors and indoors vs only indoors**	0.272	0.065	1.131	0.073
**How many scratchings**				
**>1 vs 1**	0.320	0.086	1.193	0.090
**How long the cat remains at home alone**				**0.046***
**Half day vs All day**	0.306	0.132	0.709	**0.006**
**Few hours vs All day**	0.470	0.197	1.124	**0.090**
**Never vs All day**	0.572	0.193	1.697	0.314
**Where the cat lives when he is alone**				**<0.001***
**Free indoors vs Free outdoors**	1.401	0.711	2.762	0.330
**Closed indoors vs Free outdoors**	5.405	2.322	12.585	**<0.001**
**Moving home in the last six months**				
**Yes vs No**	1.244	0.444	3.483	0.678

*overall P value

[1] not calculated because level with a few subjects

[2]Feline Immunodeficiency Virus

Table S3. Results of the bi-variable logistic regression analysis. Association between overweight and factors related to food management.

PARAMETER	Unadjusted OR	95% CI Lower	95% CI Upper	P value
**The cat eats only food proposed by the owner**				0.651*
**No, even from others vs Yes**	1.339	0.722	2.486	0.354
**No, he gets it on his own vs Yes**	-[1]	-[1]	-[1]	-[1]
**Food type**				0.487*
**Dry and wet cat food vs dry cat food**	1.429	0.798	2.558	0.230
**Homemade food vs dry cat food**	-[1]	-[1]	-[1]	-[1]
**Food quality**				0.538*
**Medium vs Low**	0.856	0.439	1.670	0.649
**High vs Low**	1.286	0.570	2.903	0.545
**Quantity of food compared with that recommended on package**				**<0.001**
**No vs Yes/yes about**	2.961	1.378	6.362	**0.005**
**I do not know vs Yes/yes about**	7.917	3.835	16.342	**<0.001**
**Use of hypocaloric food**				0.279
**Yes vs No**	1.255	0.615	2.561	0.532
**Sometimes vs No**	2.152	0.818	5.660	0.121
**Ad libitum**				
**Yes vs No**	0.707	0.397	1.258	0.238
**If ad libitum, how many times does the cat eat?**				0.099*
**3-4 times vs 1-2 times**	0.758	0.279	2.063	0.588
**>4 times vs 1-2 times**	2.247	0.681	7.409	0.184
**How many times does the cat eat?**				
**>4 times vs ≤4 times**	2.328	1.070	5.063	**0.033**
**Did you change the cat food type?**				
**Yes vs No**	0.879	0.488	1.581	0.666
**The cat asks for food**				
**Yes vs No**	0.967	0.418	2.239	0.938
**How the cat asks for food**				0.548*
**Rubs vs vocalizations**	1.448	0.678	3.092	0.339
**Paws vs vocalizations**	1.086	0.148	7.963	0.935
**Towards the food place vs vocalizations**	2.172	0.621	7.598	0.225
**The cat asks for food and then ignores it**				
**Yes vs No**	3.604	1.963	6.618	**<0.001**
**The cat extracts food from the bowl**				
**Yes vs No**	0.776	0.438	1.374	0.384
**The food bowl is near the water**				
**Yes vs No**	2.004	0.896	4.478	0.090

**Where is the bowl?**				**<0.001***
**Outdoors vs Indoors**	0.134	0.046	0.394	**<0.001**
**Both outdoors and indoors vs Indoors**	0.787	0.312	1.983	0.612
**The cat asks for food when the owner is eating**				**0.012***
**Yes vs No**	3.257	1.439	7.372	**0.005**
**Sometimes vs No**	0.963	0.497	1.866	0.910
**The owner grants extra food while sitting at the table**				
**Yes vs No**	3.559	1.827	6.934	**<0.001**
**If and how much extra food the cat gets during meals**				**0.019***
**Always vs No**	3.147	1.149	8.618	**0.026**
**Sometimes vs No**	2.073	1.035	4.151	**0.040**
**What kind of extra food**				
**Various vs Meat/ chicken / fish**	0.387	0.106	1.410	0.150
**The cat has ever changed diet?**				
**Yes/sometimes vs no**	0.875	0.414	1.849	0.726
**Fresh grass available**				
**Yes vs no**	0.226	0.097	0.524	**0.001**

*overall P value

[1] not calculated because level with a few subjects

Table S4. Results of the bi-variable logistic regression analysis. Association between overweight and factors related to playful and aggressive behaviour.

PARAMETER	Unadjusted OR	95% CI LOWER	95% CI UPPER	P value
**Play**				
**Yes vs No**	0.389	0.153	0.984	**0.046**
**Where the cat plays?**				
**Outdoors vs Indoors**	0.269	0.143	0.504	**<0.001**
**How much the cat plays with people[2]?**	1.265	0.997	1.604	0.053
**How much the cat plays with other cats[2]?**	0.893	0.742	1.074	0.230
**How much the cat plays with other animals[2]?**	0.534	0.421	0.679	**<0.001**
**How much the cat plays alone[2]?**	0.955	0.772	1.180	0.669
**How long each session of the game lasts?**				**0.047***
**2-3 min vs few seconds**	0.349	0.104	1.169	0.088
**Several minutes vs few seconds**	0.227	0.067	0.772	**0.018**
**Aggressive**				
**Yes vs No**	1.116	0.637	1.956	0.701
**If the cat is aggressive, towards whom**				0.798
**Often towards people vs sometimes towards people**	0.810	0.331	1.983	0.645
**Towards cats vs sometimes towards people**	0.622	0.140	2.757	0.532
**When the cat is aggressive?**				0.531*
**Manipulation vs Strangers**	5.000	0.511	48.909	0.167
**Animals vs Strangers**	1.042	0.402	2.697	0.933
**Unknown vs Strangers**	0.800	0.174	3.669	0.774
**Sometimes the cat seems stressed**				0.077*
**Yes vs No**	1.806	0.948	3.442	0.072
**I do not know vs no**	0.671	0.280	1.607	0.371

*overall P value

[1] not calculated because level with a few subjects

[2] associated with a score (1= never, 5= a lot) and included as a continuous variable

Table S5. Independent predictors of overweight. Results of the multivariable model

PARAMETER	Adjusted OR	95% C.I. for OR		P value
		Lower	Upper	
**Age: >7 years vs 1-7 years**	4.164	1.249	13.882	**0.020**
**Neutering: Yes vs No**	7.761	1.206	49.947	**0.031**
**Diseases**				0.981
**Urinary vs none**	0.803	0.085	7.580	0.848
**Respiratory vs none**	1.249	0.046	33.834	0.895
**Others (g/e, FIV, neoplasia, allergy, otitis) vs none**	1.478	0.147	14.825	0.740
**Where the cat lives**				0.668
**Indoor vs outdoor**	2.788	0.139	55.805	0.502
**Outdoor and indoor vs outdoor**	0.991	0.099	9.893	0.994
**Reserved space: Yes vs no**	0.286	0.081	1.009	0.052
**Number of other cohabiting animals**	0.578	0.351	0.952	**0.031**
**How long the cat remains at home alone?**				0.072
**Half day vs All day**	0.108	0.020	0.582	**0.010**
**Few hours vs All day**	0.173	0.031	0.955	**0.044**
**Never vs All day**	0.107	0.010	1.117	0.062
**Quantity of food compared with that recommended on package**				0.158
**No vs Yes/yes about**	2.263	0.462	11.091	0.314
**I do not know vs Yes/yes about**	4.465	0.971	20.532	0.055
**How many times does the cat eat?**				
**> 4 times vs ≤4 times**	1.343	0.303	5.942	0.698
**Where is the bowl?**				0.295
**Outdoor vs Indoor**	0.248	0.027	2.278	0.218
**Outdoor and Indoor vs Indoor**	0.724	0.101	5.193	0.748
**Fresh grass available: Yes vs no**	1.257	0.128	12.366	0.845
**Sometimes the cat seems stressed**				0.056
**Yes vs No**	5.117	1.204	21.750	**0.027**
**I do not know vs No**	0.540	0.049	5.956	0.615
**How much the cat plays with other cohabiting animals**[1]	1.076	.654	1.772	0.953

[1] associated with a score (1= never, 5= a lot) and included as a continuous variable
Bold values indicate significance at the p < 0.05 threshold.
