# Supplementary figures and images for: Overweight in Domestic Cats Living in Urban Areas of Italy: Risk Factors for an Emerging Welfare Issue
Source: Animals (Basel). 2021 Jul 30;11(8):2246. doi: 10.3390/ani11082246 (PMC8388420; doi:10.3390/ani11082246)

Figure S1: Feline Body Mass Index (FBMI) chart used by the researcher.

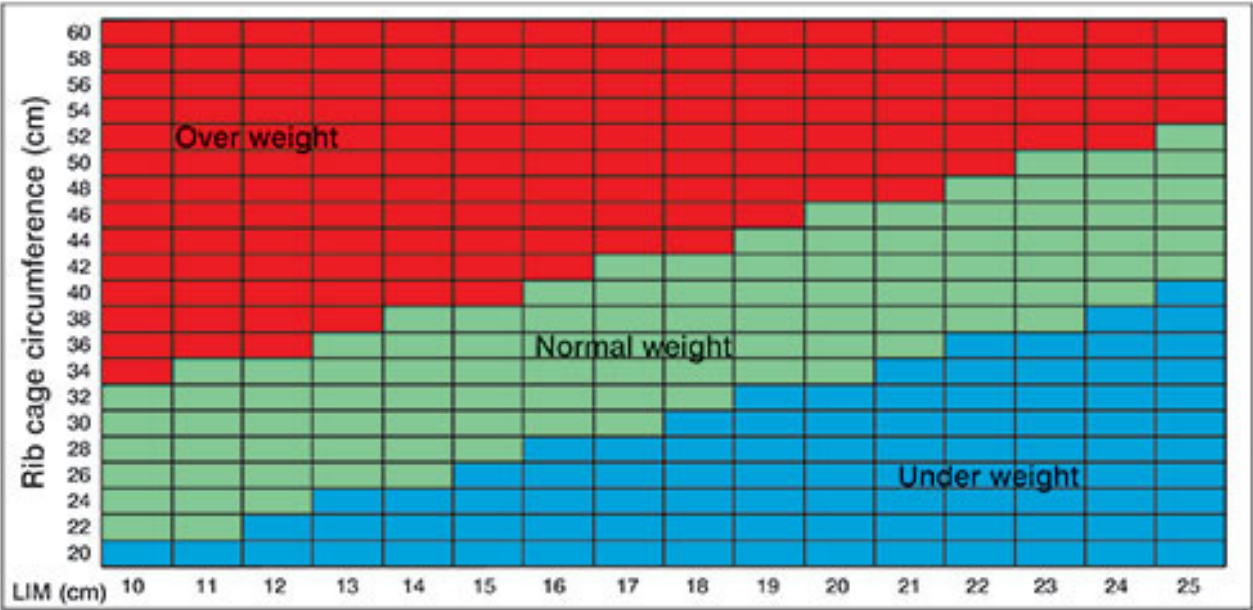

Supplement: Supplementary file 1 [file animals-11-02246-s001.zip › animals-1281905-supplementary. V2/Figure S1.pdf]
